# Supplementary material for: Estimation of stationary and non-stationary moving average processes in the correlation domain
Source: PLoS One. 2025 Jan 27;20(1):e0314080. doi: 10.1371/journal.pone.0314080 (PMC11771944; doi:10.1371/journal.pone.0314080)
Supplement: Attachment — Submitted filename: Review response.pdf [file pone.0314080.s006.pdf]

Revision notes on the manuscript PONE-D-24-28946 entitled Estimation of Moving Average Processes Using a Novel Approach to Autocorrelation Function Matching Extended to Online Estimation of Non-Stationary Processes, submitted to PLOS One

Editor:

Concern:

1. Please ensure that your manuscript meets PLOS ONE's style requirements, including those for file naming. The PLOS ONE style templates can be found at https://journals.plos.org/plosone/s/file?id=wjVg/PLOSOne_formatting_sample_main_body.pdf and https://journals.plos.org/plosone/s/file?id=ba62/PLOSOne_formatting_sample_title_authors_affiliations.pdf

Response:

The manuscript has been prepared using the official PLOS One Latex template, so we assume that manuscript meets PLOS ONE's style requirements including the references formatting. If something is not in compliance, be more specific and we will fix it.

Concern:

2. Please note that funding information should not appear in any section or other areas of your manuscript. We will only publish funding information present in the Funding Statement section of the online submission form. Please remove any funding-related text from the manuscript.

Response:

Funding-related text was removed from the manuscript and will only remain in the submission system.

Concern:

3. Thank you for stating the following financial disclosure: “This work was supported by the call for PhD students and young researchers of Slovak University of Technology in Bratislava to start a research career (Grant 23-03-01-B Impulsive Control of Biosystems).Call for PhD students and young researchers was supported by call 09I03-03-V05, financed by RRP SR.”

Please state what role the funders took in the study. If the funders had no role, please state: "The funders had no role in study design, data collection and analysis, decision to publish, or preparation of the manuscript." If this statement is not correct you must amend it as needed. Please include this amended Role of Funder statement in your cover letter; we will change the online submission form on your behalf.

Response:

The statement "The funders had no role in study design, data collection and analysis, decision to publish, or preparation of the manuscript." is fully correct.

The statement in the cover letter was modified to reflect the demand. See the updated cover letter.

Concern:

4. We note that your Data Availability Statement is currently as follows: “All relevant data are within the manuscript and in Supporting Information files.”

Please confirm at this time whether or not your submission contains all raw data required to replicate the results of your study. Authors must share the “minimal data set” for their submission. PLOS defines the minimal data set to consist of the data required to replicate all study findings reported in the article, as well as related metadata and methods (https://journals.plos.org/plosone/s/data-availability#loc-minimal-data-set-definition). For example, authors should submit the following data: - The values behind the means, standard deviations and other measures reported; - The values used to build graphs; - The points extracted from images for analysis. Authors do not need to submit their entire data set if only a portion of the data was used in the reported study. If your submission does not contain these data, please either upload them as Supporting Information files or deposit them to a stable, public repository and provide us with the relevant URLs, DOIs, or accession numbers. For a list of recommended repositories, please see https://journals.plos.org/plosone/s/recommended-repositories. If there are ethical or legal restrictions on sharing a de-identified data set, please explain them in detail (e.g., data contain potentially sensitive information, data are owned by a third-party organization, etc.) and who has imposed them (e.g., an ethics committee). Please also provide contact information for a data access committee, ethics committee, or other institutional body to which data requests may be sent. If data are owned by a third party, please indicate how others may request data access.

Response:

We confirm that the submission contains all raw data required to replicate the results of the study.

All results presented in the manuscript, including the graphs, can be fully replicated from the provided data by following the proposed methodology and using the documented parameters of the algorithms.

Concern:

5. Please remove your figures from within your manuscript file, leaving only the individual TIFF/EPS image files, uploaded separately. These will be automatically included in the reviewers’ PDF.

Response:

We do not understand why to remove the figures from the manuscript.

The figures are not within the manuscript file. The manuscript is prepared in Latex and includes individual EPS image files, which are uploaded separately. We see this as a standard procedure and not as an issue.

Concern:

6. Please include captions for your Supporting Information files at the end of your manuscript, and update any in-text citations to match accordingly. Please see our Supporting Information guidelines for more information: http://journals.plos.org/plosone/s/supporting-information.

Response:

The captions for Supporting Information files have been added at the end of manuscript and were cited in the text.

S1 Dataset Time series η for stationary process, $N = 5000$ samples (`eta_stationary_N5000.txt`)

S2 Dataset Time series η for stationary process, $N = 500$ samples (`eta_stationary_N500.txt`)

S3 Dataset $M = 100$ realizations of time series η for stationary process, $N = 5000$ samples (`eta_stationary_N5000_M100_realizations.txt`)

S4 Dataset Time series η for non-stationary process, $N = 40000$ samples (`eta_nonstationary.txt`)

S5 Dataset $M = 100$ realizations of time series η for non-stationary process, $N = 40000$ samples (`eta_nonstationary_N40000_M100_realizations.txt`)

Concern:

7. We are unable to open your Supporting Information file data_offline_N500.mat, data_offline_N5000.mat and data_online.mat. Please kindly revise as necessary and re-upload.

Response:

Supporting Information files data_offline_N500.mat, data_offline_N5000.mat and data_online.mat. were originally uploaded in the form of Matlab data files contain the workspace variables for the individual experiments. Since this is not appropriate, we will upload the data in more universal format (txt). Each file now contains a timeseries (sequence) of input or output noise and is named accordingly. See the re-uploaded data.

Reviewer 1:

Concern:

The title is dense, it needs simplification or rephrasing to enhance clarity and impact.

Response:

We simplified the title as follows:

Estimation of stationary and non-stationary moving average processes in the correlation domain

Concern:

The novelty of the approach and key findings need to be emphasized in the abstract.

Response:

We rewritten the abstract to highlight the novelty and key findings as:

This paper introduces a novel approach for the offline estimation of stationary moving average processes, further extending it to efficient online estimation of non-stationary processes. The novelty lies in a unique technique to solve the autocorrelation function matching problem leveraging that the autocorrelation function of a colored noise is equal to the autocorrelation function of the coefficients of the moving average process.

This enables the derivation of a system of nonlinear equations to be solved for estimating the model parameters. Unlike conventional methods, this approach uses the Newton-Raphson and Levenberg–Marquardt algorithms to efficiently find the solution.

A key finding is the demonstration of multiple symmetrical solutions and the provision of necessary conditions for solution feasibility. In the non-stationary case, the estimation complexity is notably reduced, resulting in a triangular system of linear equations solvable by backward substitution. For online parameter estimation of non-stationary processes, a new recursive formula is introduced to update the sample autocorrelation function, integrating exponential forgetting of older samples to enable parameter adaptation. Numerical experiments confirm the method's effectiveness and evaluate its performance compared to existing techniques.

Concern:

While various methods are mentioned in the introduction section, the motivation for choosing the specific approach should be highlighted more explicitly.

Response:

We understand the concern. The motivation for pursuing the proposed approach was highlighted explicitly with respect to other relevant methods.

The manuscript was modified as follows:

This paper addresses the problem of moving average processes estimation in the correlation domain rather than in the time domain as in the case of prediction-error methods.

One of the motivations for pursuing the approach presented in this paper, compared to other reviewed methods, is its recursive formulation of the parameter estimation problem. This allows for an efficient update of the parameter estimate with each new sample of correlated noise. Additionally, it minimizes the computational complexity of online estimation to the solution of a triangular system of linear equations, eliminates the nonlinear effects typical of pseudolinear regression, enables the estimation of the first model coefficient, which is not possible within the prediction-error methods and removes the need to find and numerically maximize the likelihood function of colored noise.

Concern:

Clearly state the assumptions made in the derivations (e.g., ergodicity, properties of the input noise).

Response:

The crucial theoretical/mathematical assumptions are now clearly and explicitly stated so they cannot be overlooked. There are no other assumptions than the basic properties of the white noise.

The manuscript was modified as follows:

Materials and methods

Moving average process

Consider the moving average process defined as [1, 15, 16]

$$\epsilon_{(k)} = g(z)\eta_{(k)} \ , \tag{1}$$

where $\eta \sim \mathcal{N}\left(0, \sigma_\eta^2\right)$ is the uncorrelated zero-mean white noise input and ϵ represents the correlated colored noise output.

The input noise $\eta_{(k)}$ is assumed to be ergodic and to satisfy

$$\begin{aligned} &E\left\{\eta_{(k)}\right\} = 0 \quad \forall k \ , \\ &E\left\{\eta_{(k)}^2\right\} = \sigma_\eta^2 \quad \forall k \ , \\ &E\left\{\eta_{(k)}\eta_{(k+i)}\right\} = 0 \quad \forall k \ , \forall i \neq 0 \ . \end{aligned} \tag{2}$$

Concern:

Consider discussing how the choice of the forgetting factor (λ) affects the results and any guidelines for selecting it.

Response:

We accept the concern.

However, some tuning rules were already mentioned in the original version of the manuscript.

The paragraph describing the tuning of the forgetting factor was extended as follows:

The forgetting factor λ in Eq (26) is considered the tuning parameter, which should be empirically chosen with a regard to the anticipated statistical properties of the estimated process. Higher values of λ (approaching 1) should correspond to close-stationary processes with parameters changing very slowly over time, whereas lower values should be used for the estimation of non-stationary processes, the parameters of which are evolving rather rapidly. It can be claimed that the choice of λ represents a trade-off between the adaptation rate and the variance of the parameter estimate. A lower λ allows for faster adaptation but induces the undesired phenomenon of forgetting useful information comprised in older samples of the dataset causing rapid fluctuations in the estimated parameters and making it more sensitive to outliers in data. A higher λ provides more robust and less volatile parameter estimate, but may result in sluggish response to the actual changes in parameters of moving average process.

Besides that, there were added experimental results analyzing the effect of forgetting factor.

Fig 14 then shows that the adaptation of parameters was faster than in Fig 12, but their volatility (uncertainty) also increased significantly, which is undesired. In contrast, Fig 15 demonstrates slower and less volatile adaptation of the estimated parameters.

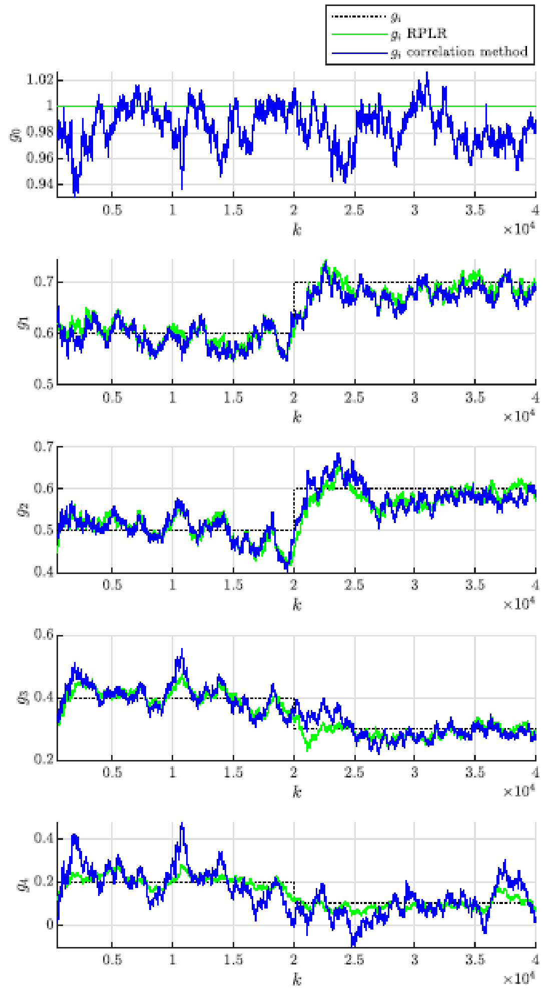

Fig 14. Online estimation of coefficients $g_i(k)$ of the non-stationary moving average process as a function of number of processed samples k obtained assuming $\lambda = 0.999$ using the proposed correlation method and the recursive pseudolinear regression approach

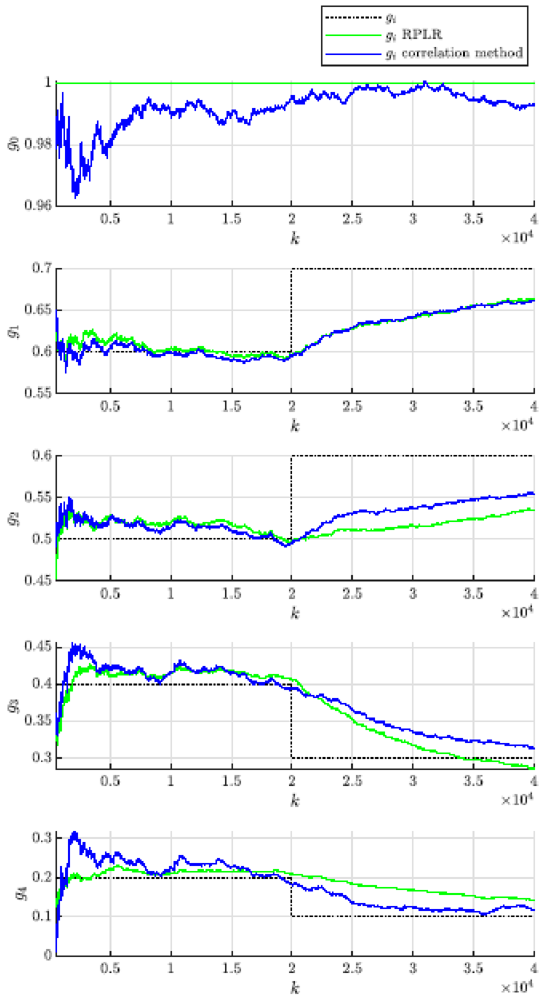

Fig 15. Online estimation of coefficients $g_i(k)$ of the non-stationary moving average process as a function of number of processed samples k obtained assuming $\lambda = 0.99995$ using the proposed correlation method and the recursive pseudolinear regression approach

Concern:

Discuss some limitations or potential areas for future research to provide a more balanced view.

Response:

We accept the concern. The limitations and directions for the future research of the proposed method are now discussed in the conclusions as follows:

Despite the advantages, several limitations and shortcomings have been identified that need to be acknowledged. While we analyzed the impact of the forgetting factor λ on the parameter estimate, determining the optimal λ in practice can be challenging, especially in problems where the statistical properties change rapidly. The existence of four symmetrical solutions to the estimation problem means that four different models share the same autocorrelation function, which may lead to ambiguity. The convergence of the algorithm is sensitive to the choice of the initial guess; a poor choice of the initial guess may result in slow convergence or convergence to a symmetrical solution. Finally, the derived feasibility condition means that for an inappropriate sample autocorrelation function, which may be a result of insufficient data quantity, the estimation problem might not have a solution.

These findings suggest the following possible directions for the future research. Future research should explore ways to reliably select the correct solution from the set of possible symmetrical solutions. This could involve applying regularization techniques that favor specific solutions based on prior knowledge or model assumptions. Another option is implementing an adaptive forgetting factor λ that automatically adjusts based on the data's characteristics, which could improve the performance of the method.

Reviewer 2:

Concern:

One of the main concerns with the article is the lack of sufficient experimental validation to support the claim that the proposed method is suitable for real-time applications. Although the capability for online estimation is mentioned, there are no robust experiments demonstrating that the algorithm can operate in real-time scenarios with latency constraints and continuous processing. The authors should include experiments using real-time data to analyze the method's ability to adapt to rapid parameter changes under real-world, real-time conditions.
This leads to a second observation: the proposed method has been tested primarily in controlled environments and simulations with synthetically generated data. There is no evaluation of how the method behaves in the presence of real-world noise or under less ideal conditions common in practical applications.
It is recommended to:
- Include tests with real datasets that introduce noise, missing data, or nonlinearities that could affect parameter estimation.
- Conduct a sensitivity analysis of the method with respect to different levels of noise and data variations to demonstrate that the algorithm is stable and capable of handling real-world situations.

Response:

First there needs to be clarified that we inappropriately used term "real-time" three times in the manuscript because the method was actually never meant to be used for real-time systems, which involves dealing with latency constraints and continuous processing specific for real-time systems. Therefore, the incorrect terminology "real-time" was removed from the paper.

What really characterizes the proposed method is term online estimation, which means that the parameter estimate is updated each iteration using the new available sample of colored noise, rather than using the offline approach where all gathered samples are processed at once.

Second, we agree that the proposed method has been tested primarily in controlled environments and simulations with synthetically generated data. We also accept the recommendations to include tests with real datasets to demonstrate that the algorithm is stable and capable of handling real-world situations. However, there needs to be mentioned that in the case of real-world data in the form of colored noise sequences, the actual parameters of the underlying moving average process are always unknown, hence accuracy analysis by comparing the model coefficients is simply not possible. This fact is not that discouraging because we can still test the algorithm ability to handle real data and at least compare the results with the recursive pseudolinear regresssion, so this experimentation will be carried out.

The following experimentation was added to the manuscript.

Note that we also tested different sequences from this dataset with similar results; however, including visual representations of these results as graphs would significantly increase the manuscript's length, which is already at 36 pages. Besides, many noise signals in the dataset featured relatively low frequencies (with respect to the sampling frequency), which ultimately required to consider higher model order making a neat visualization of the results difficult.

To demonstrate that the proposed estimation algorithm can handle real-world data in the form of sampled colored noise sequences, rather than only artificially generated ones, further experimentation will be conducted. Real data involving a correlated (colored) noise sequence, which represents a realization of some non-stationary moving average process with unknown coefficients, typically contain adverse features such as nonlinearities, varying noise levels, and nonzero mean that may impact parameter estimation. However, it should be noted that in this case, the actual parameters of the underlying moving average process are completely unknown; therefore, directly assessing the accuracy will not be possible.

For this experiment, we will work with the publicly available Environmental Sound Classification dataset (ESC-50) [29]. The ESC-50 dataset is a labeled collection of audio recordings originally designed for testing methods of sound classification, spectral analysis, and signal processing in general. It comprises 2000 five-second clips across 50 different classes, including natural, human, and domestic sounds. The most relevant types of sounds for this experiment, which naturally exhibit the characteristics of non-stationary colored noise, are wind, sea waves or rainfall. These natural noise sounds have time-dependent spectra and non-stationary statistical properties that evolve throughout the duration of the sampled sound sequence. The practical aim of the experiment is to determine the evolution of the coefficients of the underlying moving average process that produces this sound, i.e., to determine the mathematical model of the noise. The database of samples was downloaded from the publicly available repository `https://github.com/karolpiczak/ESC-50`.

The considered sampling frequency $f_s = 22500$ Hz and the duration of each recording for 5 seconds implies the total number of samples $N = 110250$. For the colored noise from the recording *2-81731-A-10.wav* (rainfall), the model order was chosen as $n_g = 5$ based on the prior assessment of the autocorrelation function, and the forgetting factor λ was carefully tuned to obtain the optimal adaptation rate as $\lambda = 0.9995$. The envelope of the colored noise signal $\epsilon_{(k)}$ from the recording *2-81731-A-10.wav* visualized in Fig 17 suggest that this signal features highly variable noise levels.

The resulting evolution of the estimated model parameters with respect to the sample number k using the proposed correlation method and the RPLR is shown in Fig 18. This figure documents relatively large and abrupt fluctuations of the parameters, while this primarily correlates with the volatility of the signal amplitude (envelope), which translated into simultaneous change of all model coefficients. It is also noticeable that the RPLR provided different results, as it is not capable of estimating models with varying static gain (variable noise levels) because the method assumes the first model coefficient is fixed as $g_0 = 1$.

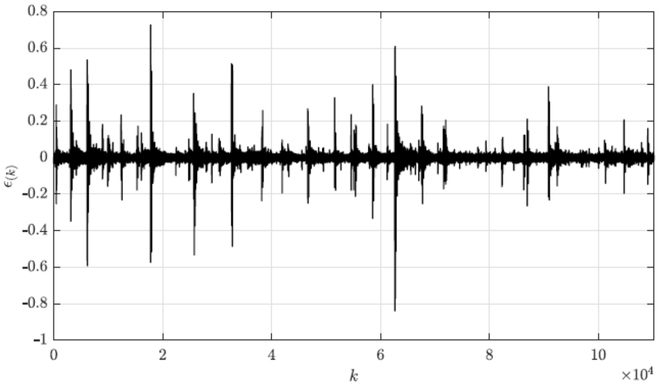

Fig 17. Envelope of the colored noise signal $\epsilon_{(k)}$ from recording *2-81731-A-10.wav* as a function of sample number k

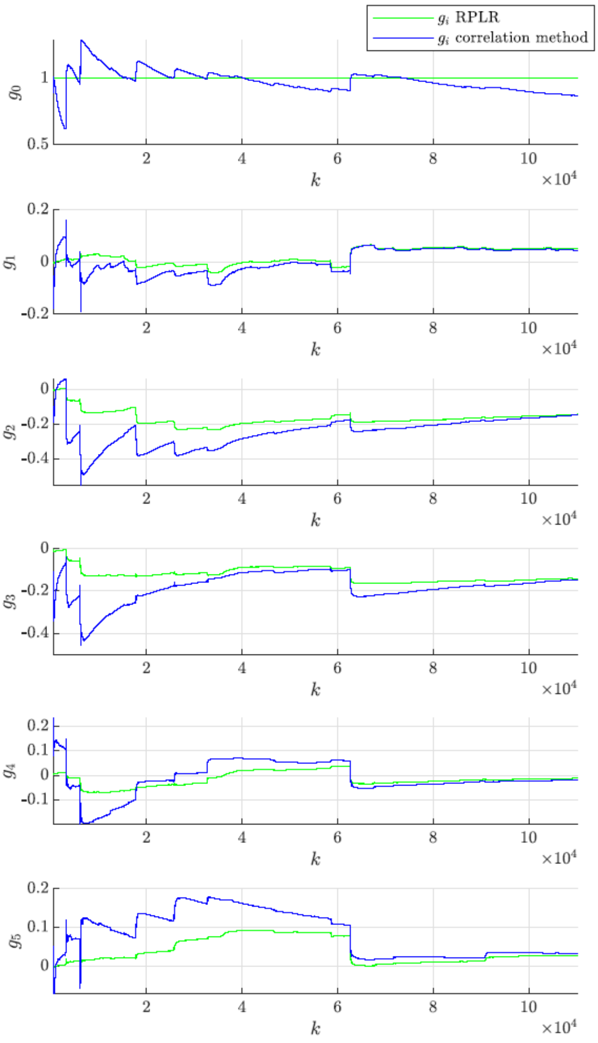

Fig 18. Online estimation of the coefficients $g_i(k)$ of the non-stationary moving average process based on the colored noise data from recording *2-81731-A-10.wav* as a function of sample number k obtained using the proposed correlation method and the recursive pseudolinear regression approach

Here are the additional demonstrations which do not fit into the manuscript mainly because they are not interesting/different to show some important property:

For signal 4-167063-C-11 we obtained:

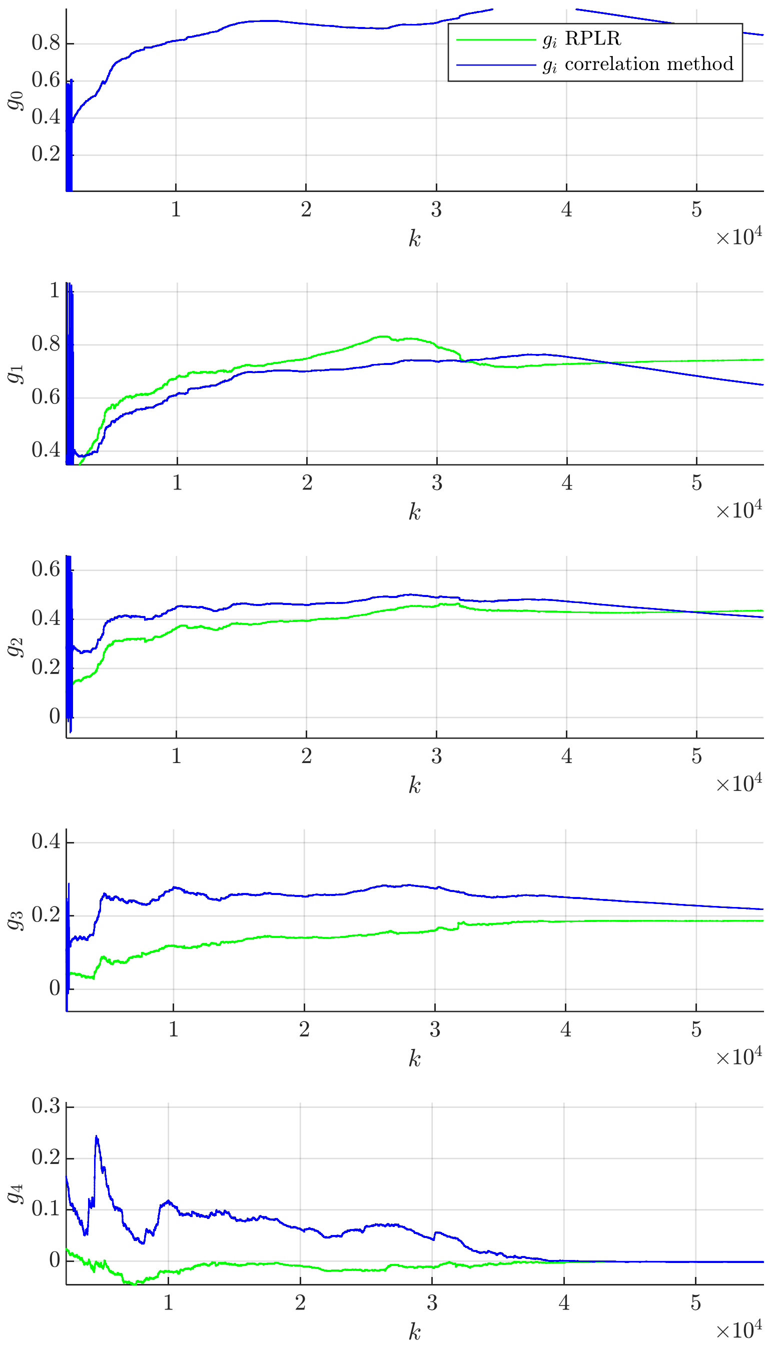

For signal 1-56311-A-10 we obtained:

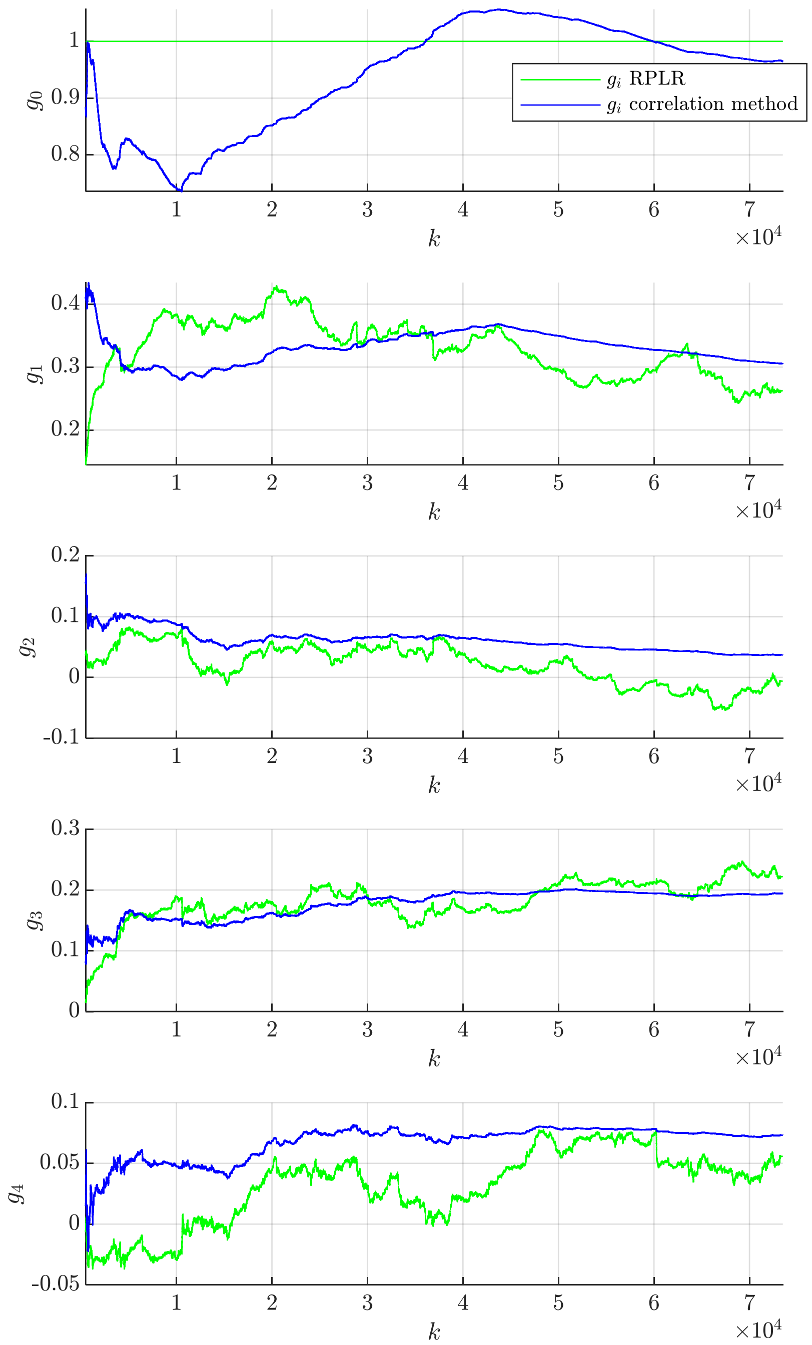

For signal 1-21189-A-10 we obtained:

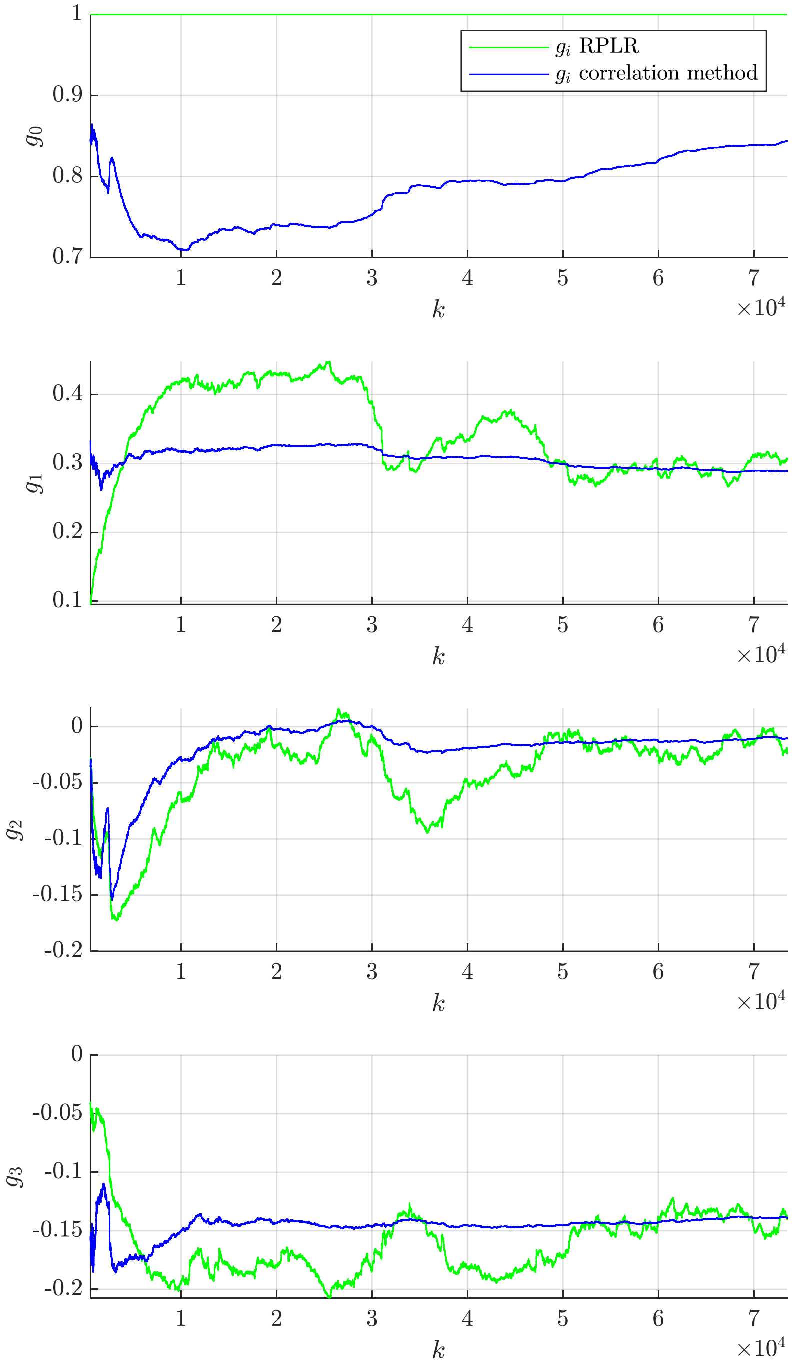

g_i RPLR
g_i correlation method
g_0
g_1
g_2
g_3
k
$\times 10^4$

Concern:

Furthermore, the article makes strong claims about the reduction of computational complexity of the methodology, especially in the context of online estimation of non-stationary processes. However, a detailed analysis of the algorithm's time and space complexity is not provided, nor is there a quantitative comparison with other methods. It is advisable to compare the performance of the proposed method with other techniques, such as pseudolinear regression and prediction error-based methods, through quantitative benchmarking experiments.

Response:

We understand and accept the concern. The following analysis and benchmarking experiments were conducted and added to the manuscript.

Analysis of the proposed method:

Computational complexity and memory footprint analysis

Now analyze the overall memory footprint and per-iteration computational complexity of the proposed correlation-based method for online estimation of non-stationary process.

Based on the linear equation system given by Eq (46), it can be concluded that there needs to be stored maximally n_g model coefficients estimated from n_g previous algorithm iterations, which implies $\frac{n_g(n_g+1)}{2}$ variables. Moreover, the current sample autcorrelation functions $\hat{R}_{\epsilon\epsilon}(n,k)$ and the values of individual summations $s(n,k)$ need to be stored for arguments $n = 0 \ldots n_g$, which results in additional $2(n_g+1)$ real-valued variables. To recursively update the sample autocorrelation function according to Eq (26) for $n = 0 \ldots n_g$, there needs to be stored $n_g + 1$ past values of the colored noise $\epsilon_{(k-n)}$. Summarizing the above gives the minimal memory footprint of $\frac{n_g(n_g+1)}{2} + 3(n_g+1)$ real-valued variables.

Concerning the computational complexity per algorithm iteration, recursively updating the individual summations $s(n,k)$ for $n = 0 \ldots n_g$ according to (25) represents a linear complexity $n_g + 1$, and the recursive update of the sample autocorrelation function $\hat{R}_{\epsilon\epsilon}(n,k)$ for arguments $n = 0 \ldots n_g$ according to Eq (26) also has a linear complexity $n_g + 1$. Solving the triangular system of linear equations given by Eq (46) according to Eq (47) can be achieved with a reduced quadratic complexity of $\frac{n_g(n_g-1)}{2}$. Finally, the last computation given by Eq (48) can be achieved with linear complexity n_g. Considering big O notation, the asymptotic computational per-iteration complexity of this algorithm is $\mathcal{O}\left(n_g^2\right)$.

Analysis of the recursive pseudolinear regression:

Based on the equations above, the following conclusions can be drawn about the computational complexity per iteration and the overall memory footprint of the recursive pseudolinear regression. To store $P(k)$, $P(k-1)$, $Y(k)$, $\hat{\theta}(k)$, $h(k)$ there are required at least $2n_g^2 + 3n_g$ real-valued variables. It is a known characteristic of the recursive least squares method that not all updates given by Eq (56) can be performed "in place" without requiring an additional memory allocation for temporary variables and intermediate calculations, so the actual memory footprint is even larger. Therefore, it is apparent that the total memory footprint exceeds that of the proposed method, which was analyzed and discussed earlier.

Concerning the computational complexity of the recursive least squares update given by Eq (56), linear operations including the matrix-vector multiplications, inner and outer products, as well as scalar multiplications and element-wise matrix additions are required. Due to multiple matrix operations with quadratic time complexity, the resulting complexity will be higher than that of the correlation-based method, which was shown to have only one quadratic term. However, considering big O notation, the asymptotic computational complexity of this algorithm is still quadratic $\mathcal{O}\left(n_g^2\right)$.

Experimental evaluation:

Finally, a quantitative benchmarking experiment will be conducted to compare the computational efforts required for a single iteration of the proposed correlation method and the recursive pseudolinear regression method in the case of online estimation of non-stationary processes. To ensure a fair comparison when measuring the execution time of a single iteration (runtime), we will use a dedicated Matlab function, *timeit*, which runs the tested functions multiple times automatically to provide a more stable and averaged measurement of the execution time. This helps reduce the impact of anomalies, such as background processes consuming CPU resources, during the benchmark. It is important to note that careful implementation of both methods was vital to avoid potentially biased results that could favor one method over the other only due to slight programming differences affecting the overall performance.

The resulting dependency of the average execution time per iteration for the compared methods, as a function of the number of estimated parameters n_g (the order of the moving average model), is shown in Fig 19. This figure demonstrates that the recursive pseudolinear regression method is slower than the correlation-based method, which provides at least 2x faster iteration execution. This experiment also confirms that both the proposed correlation method and the recursive pseudolinear regression have quadratic asymptotic time complexity; however, the latter has a larger constant growth factor (gain). It is important to note that the exact ratio between these complexities (runtimes) also depends on the programming language, compiler, and hardware platform. Nevertheless, the correlation-based method will generally outperform the recursive pseudolinear regression due to the lower number of operations with quadratic time complexity and reduced nested for loop resulting from back substitution in Eq (47).

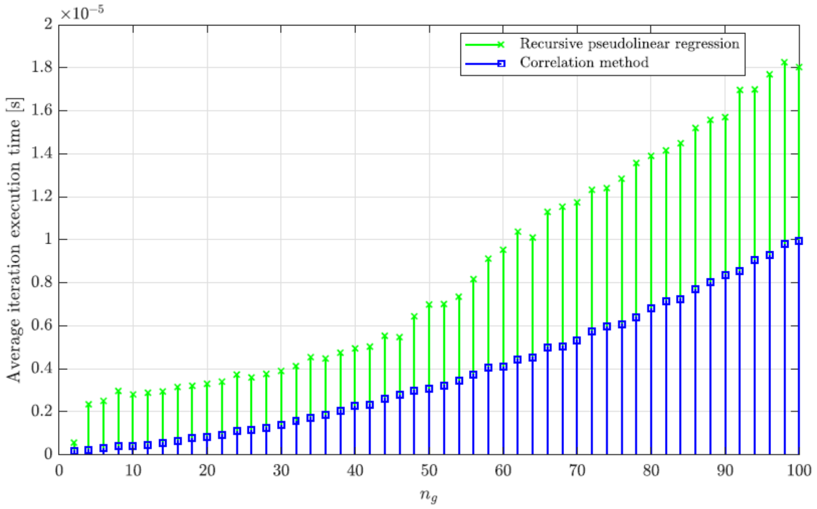

Fig 19. Dependency of the average execution time of a single iteration corresponding to the proposed correlation method and the recursive pseudolinear regression as a function of the number of estimated parameters n_g

The conclusion was also modified to not make such strong claims:

> Given these points, the proposed correlation method emerges as a compelling alternative to traditional prediction-error methods as it offers comparable or even better estimate accuracy and lower computational complexity, depending on the scenario and the problem dimension.

Concern:

Finally, although the proposed method is shown to outperform other methods in terms of estimation accuracy, the inclusion of additional statistical tests—such as hypothesis tests or significance analyses—would help to more rigorously validate that the observed differences between methods are statistically significant and not simply a product of data variability.

Response:

We understand and accept the concern. It is truly beneficial to perform further statistical analysis of the estimation accuracy of all the compared methods. We acknowledge that the estimation accuracy was originally tested/compared considering only a single experiment (one realization of input white noise), without repeating and validating the estimation with different randomly generated data.

Therefore, we decided to perform the following: The presented estimation experiments, including the stationary and non-stationary processes, which considered synthetically generated input white noise and output correlated noise, were repeated multiple times with different randomly generated noise sequences (multiple realizations), and evaluated to obtain the corresponding parameter estimates. The use of experimental data in the case is unfeasible due to the data quantity and the need for repeating the same statistical conditions many times (controlled conditions). To assess the results, the mean values and the variances of the estimated parameters residuals were obtained from these results. Based on them, the claims about the estimation accuracy can be rigorously supported. We

think that the presented evaluation of the statistical properties of the parameter estimate residuals is more insightful and valuable than the hypothesis testing.

The following was added to the manuscript:

The presented experiments, which involve data in the form of randomly generated noise sequences of length $N = 5000$ samples, were repeated for multiple (M) different realizations of the random white noise sequences η. These realizations yielded the corresponding correlated noise sequences ϵ, which were used for model estimation. For the i-th of the total $M = 100$ realizations, the parameter estimate vector $\mathbf{g}_i^*$ was determined using each of the three compared methods (correlation, Durbin's and pseudolinear regression), and the corresponding statistics were then evaluated. To assess the performance in the statistical framework, the mean values and the sample variances of the residuals of the estimated parameters were calculated.

The mean value vector of the residuals vector $\mathbf{g}_i^* - \mathbf{g}$ was obtained as

$$\mu_{(\mathbf{g}^*-\mathbf{g})} = \frac{1}{M}\sum_{i=1}^{M}\left(\mathbf{g}_i^* - \mathbf{g}\right) . \tag{53}$$

The vector of variances of the residuals vector $\mathbf{g}_i^* - \mathbf{g}$ was obtained as

$$S^2_{(\mathbf{g}^*-\mathbf{g})} = \frac{1}{M}\sum_{i=1}^{M}\operatorname{diag}\left(\left(\mathbf{g}_i^* - \mathbf{g}\right)^{\mathrm{T}}\left(\mathbf{g}_i^* - \mathbf{g}\right)\right) . \tag{54}$$

The motivation for conducting the statistical analysis described above is to rigorously assess the performance of the compared methods using multiple random realizations of noise sequences, thereby supporting the validity of the results. The results for the stationary process (49) summarized in Table 2 show that the proposed correlation method provided mean values of the residuals $\mu_{(\mathbf{g}^*-\mathbf{g})}$ closest to zero, while the corresponding variances of the residuals $S^2_{(\mathbf{g}^*-\mathbf{g})}$ were similar to those obtained using Durbin's method. Thus, it can be claimed that their performances are very similar in terms of bias and efficiency. In contrast, it can be concluded that the pseudolinear regression performed significantly worse, resulting in both larger mean values and larger variances of the residuals.

	$\mu_{(\mathbf{g}^*-\mathbf{g})}$
Correlation method	$(-2.8\times10^{-3}\ -5.1\times10^{-3}\ -3.2\times10^{-3}\ 1.5\times10^{-3}\ -1.8\times10^{-3}\ -2.9\times10^{-3})^{\mathrm{T}}$
Durbin's method	$(5.8\times10^{-3}\ -8.1\times10^{-3}\ -6.7\times10^{-3}\ -2.8\times10^{-3}\ -28.2\times10^{-3}\ -0.4\times10^{-3})^{\mathrm{T}}$
Pseudolinear regression	$(61.6\times10^{-3}\ -52.1\times10^{-3}\ -117.5\times10^{-3}\ -146.3\times10^{-3}\ -177.4\times10^{-3}\ -190.6\times10$[illegible]
	$S^2_{(\mathbf{g}^*-\mathbf{g})}$
Correlation method	$(0.2\times10^{-3}\ 0.7\times10^{-3}\ 0.6\times10^{-3}\ 0.8\times10^{-3}\ 0.8\times10^{-3}\ 2.6\times10^{-3})^{\mathrm{T}}$
Durbin's method	$(0.2\times10^{-3}\ 0.7\times10^{-3}\ 0.6\times10^{-3}\ 0.7\times10^{-3}\ 1.6\times10^{-3}\ 0.8\times10^{-3})^{\mathrm{T}}$
Pseudolinear regression	$(4.2\times10^{-3}\ 3.3\times10^{-3} 14.3\times10^{-3}\ 22.0\times10^{-3}\ 32.1\times10^{-3}\ 37.2\times10^{-3})^{\mathrm{T}}$

Table 2. Statistical evaluation of the estimation accuracy of the stationary moving average process by the sample mean $\mu_{(\mathbf{g}^*-\mathbf{g})}$ and sample variance $S^2_{(\mathbf{g}^*-\mathbf{g})}$ of the estimate residuals $\mathbf{g}_i^*-\mathbf{g}$ for $M = 100$ experiment realizations and $N = 5000$ samples

A similar experimental evaluation was conducted also for the online estimation of the non-stationary process. However, since the estimated parameters are in the process of adaptation (transition), there is no reason to determine their variances at each sample. Instead, the mean values of the estimated parameters as a function of the sample number are evaluated across multiple realizations of the input while noise, which provides more robust assessment of the estimation accuracy in the case of nonstationary processes.

The following was added to the manuscript:

The online estimation of the non-stationary process given by Eq (55) was repeated for multiple (M) different realizations of the random input white noise sequences η. The corresponding timeseries of η are in S5 Dataset. For the i-th of the total $M = 100$ realizations, the parameters were determined as functions of the sample number k using the proposed correlation method and the RPLR, as described above. This statistical approach mitigates the influence of a realization of the input noise sequence η on the parameter estimate, thereby enabling a more robust assessment of the estimation performance. To this end, the mean values of the estimated parameters as functions of the sample number k were evaluated across M realizations of the input noise sequence and are plotted in Fig 16. This figure shows that the proposed correlation method provides surprisingly fast initial adaptation and, more importantly, the estimate converges to the actual parameters with little to no statistical bias, compared to the RPLR, which appears to have significant bias in the fourth and the fifth parameter.

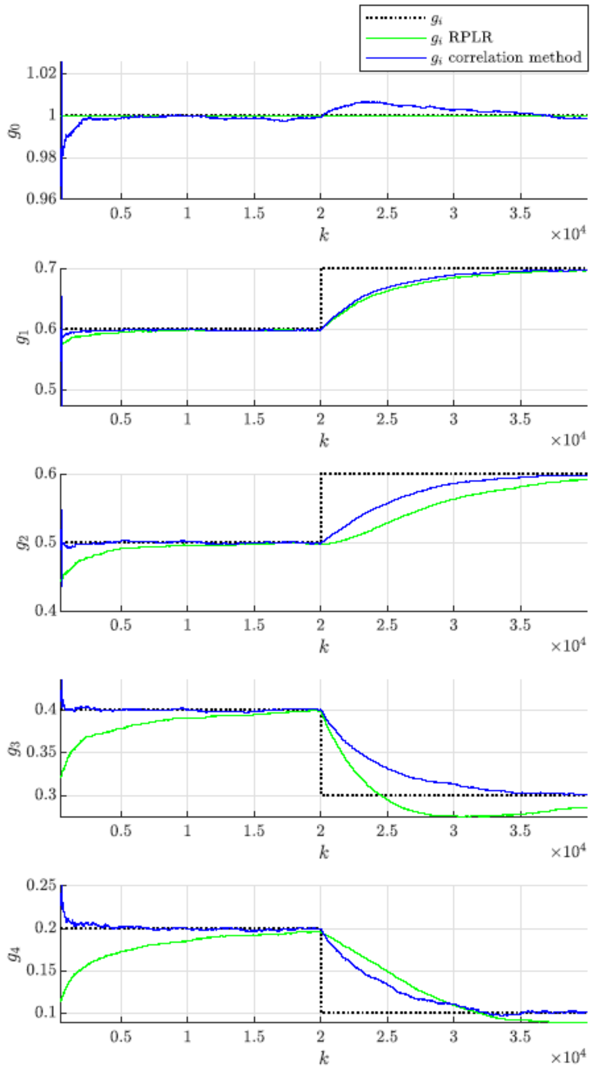

Fig 16. Mean values of the estimated of the coefficients $g_i(k)$ of the non-stationary moving average process as a function of sample number k obtained assuming $\lambda = 0.99998$ using the proposed correlation method and the RPLR across $M = 100$ realizations of input noise sequence

The conclusion was modified accordingly.

The presented numerical experiments confirmed that the proposed method can provide estimates of stationary and non-stationary moving average processes with the accuracy comparable, or even superior to the conventional prediction-error methods. The statistical evaluation, considering multiple realizations of the input noise sequence, demonstrated that the correlation-based method performed similarly to Durbin's method and significantly outperformed the pseudolinear regression in terms of statistical bias and efficiency. It can be concluded that the accuracy of the parameter estimate is related to the variance of the sample autocorrelation function, which is determined by the number of processed samples and by the forgetting factor.
